# Intrinsic and Extrinsic Factors Affecting Microtubule Dynamics in Normal and Cancer Cells

**DOI:** 10.3390/molecules25163705

**Published:** 2020-08-14

**Authors:** Filip Borys, Ewa Joachimiak, Hanna Krawczyk, Hanna Fabczak

**Affiliations:** 1 Laboratory of Cytoskeleton and Cilia Biology Nencki Institute of Experimental Biology of Polish Academy of Sciences, 3 Pasteur Street, 02-093 Warsaw, Poland; fborys@ch.pw.edu.pl; 2 Department of Organic Chemistry, Faculty of Chemistry, Warsaw University of Technology, 3 Noakowskiego Street, 00-664 Warsaw, Poland; hkraw@ch.pw.edu.pl

**Keywords:** α-tubulin, β-tubulin, microtubule, post-translational modifications, cancer, microtubule-targeting agents (MTAs), tubulin-binding agents (TBAs), resistance

## Abstract

Microtubules (MTs), highly dynamic structures composed of α- and β-tubulin heterodimers, are involved in cell movement and intracellular traffic and are essential for cell division. Within the cell, MTs are not uniform as they can be composed of different tubulin isotypes that are post-translationally modified and interact with different microtubule-associated proteins (MAPs). These diverse intrinsic factors influence the dynamics of MTs. Extrinsic factors such as microtubule-targeting agents (MTAs) can also affect MT dynamics. MTAs can be divided into two main categories: microtubule-stabilizing agents (MSAs) and microtubule-destabilizing agents (MDAs). Thus, the MT skeleton is an important target for anticancer therapy. This review discusses factors that determine the microtubule dynamics in normal and cancer cells and describes microtubule–MTA interactions, highlighting the importance of tubulin isoform diversity and post-translational modifications in MTA responses and the consequences of such a phenomenon, including drug resistance development.

## 1. Introduction

**Figure 1:**
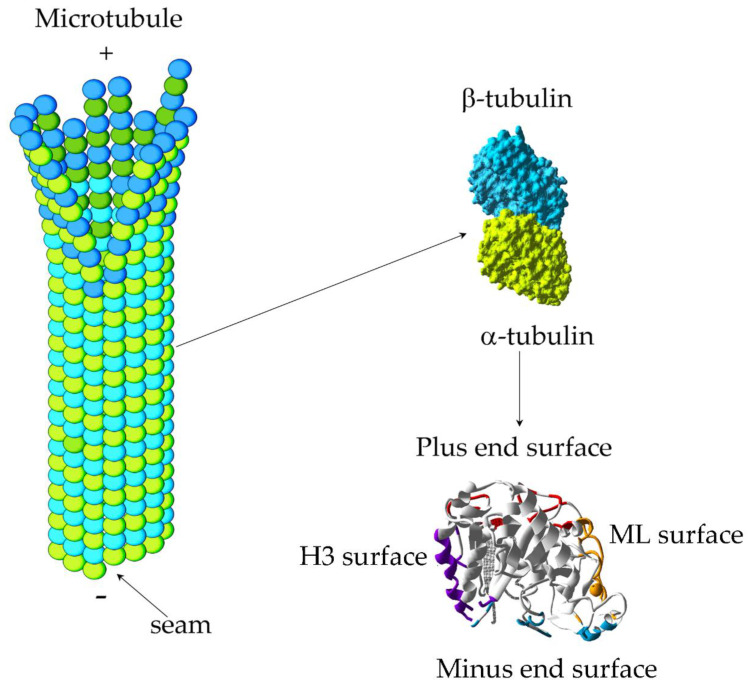
Scheme of the structure of microtubules, tubulin heterodimers, and functional surfaces of tubulin.

Microtubules (MTs), which are dynamic cytoskeletal components, are hollow-tube filaments usually built up of 13 protofilaments composed of α- and β-tubulin heterodimers connected by noncovalent bonds (Figure 1). In eukaryotic cells, the initiation of tubulin polymerization, so-called nucleation, is associated with the presence of templates, including the γ-tubulin ring complex (γ-TuRC) or newly severed microtubule ends (so-called seeds) [1]. The elongation of polymerizing microtubules results in the formation of a microtubular network which is involved in many cellular processes, such as cell movement, cell shape determination, distribution of organelles, intracellular transport, and the formation of the mitotic spindle, the key structure in chromosome segregation during cell division [2,3]. Moreover, microtubules form skeletons of complex eukaryotic structures such as centrioles, basal bodies, and cilia [4]. Not surprisingly, defects in microtubule assembly or properties can lead to severe diseases, including cancer [5].

Performing such different functions requires both the stability of microtubules and the ability to quickly respond to cellular cues by shortening or directional growth. Within the cell, microtubule dynamics depends on three intrinsic factors: (i) the isotype of the incorporated α- and β-tubulins (as in most organisms, tubulins are encoded by multiple genes) [6,7,8,9,10]; (ii) the type and level of post-translational tubulin modifications [11,12]; and (iii) interactions with non-tubulin proteins, so-called microtubule-associated proteins (MAPs) [13,14]. Besides the intrinsic factors that generate heterogeneous MTs, extrinsic physical factors (e.g., temperature) and chemical factors, so-called microtubule-targeting agents (MTAs; also known as tubulin-binding agents (TBAs), microtubule-interfering drugs, anti-microtubule drugs, or microtubule poisons) can influence MT dynamics. Taking advantage of the possibility to externally modulate MT dynamics, several microtubule-targeting agents have been employed as chemotherapeutic agents [15,16,17].

The effect of an individual anti-tubulin/anti-microtubule compound on the polymer mass, stability, and dynamics of microtubules is very complex. Basically, MTAs can be divided into two main categories. Compounds of the first category (microtubule-stabilizing agents (MSAs)), after binding to the tubulin heterodimer, increase the lateral interactions between heterodimers, which at high compound concentration leads to increased polymerization and stabilization of MTs, resulting in increased polymer mass within the cell. Compounds from the second group (microtubule-destabilizing agents (MDAs)) decrease or inhibit mainly longitudinal interactions between heterodimers at high concentrations, resulting in MT depolymerization and decreased polymer mass. Notably, at lower concentrations used in clinical applications, both types of MTAs only finely tune MT dynamics, with no effect on polymer mass or overall cytoskeleton features [16,18]. To date, six binding sites are recognized in the tubulin surface and numerous small molecules or macrocyclic compounds that alter intra- and intertubulin interactions by binding with these pockets [18].

In this review, we focus on the factors that determine microtubule dynamics in normal and cancer cells. We describe the microtubule–MTA interplay, highlighting the significance of the diversity of tubulin isoforms and their post-translational modifications in MTA response, and the consequences of this phenomenon, including the development of drug resistance. Due to the large number of chemical compounds used in cancer therapies, this paper covers only a narrow range of molecules.

## 2. Structure of Tubulin: Molecular Basis of MT Dynamics

The 3D structure of microtubule subunits was resolved in 1998 [19]. That study and subsequent studies showed that α- and β-tubulins share high structural similarity; both are composed of several large secondary structures, including 10 β-strands (S1–S10) and 12 α-helices (H1–H12) linked by loops [19]. Additionally, six small helices (H1’, H2’, H2”, H3’, H9’, and H11’) are located on loops near the larger structures [20]. Helices H1–H12 and strands S1–10 constitute the protein body, which can be divided into three main domains: an N-terminal, also called a nucleotide-binding domain, a so-called intermediate domain, and a C-terminal part. The N-terminal domain (amino acids 1 to ~205) comprises six parallel β-strands (S1–S6) alternating with α-helices (H1–H7) linked by loops. The intermediate domain (aa ~206–381) is formed by three helices (H8–H10) alternating with four β-strands (S7–S10). The C-terminal domain (aa ~382 to ~451) is composed of two α-helices (H11–H12), followed by several unstructured amino acids that extend beyond the globular part as the so-called tubulin tail [19,20].

The nucleotide-binding and intermediate domains form the main part of the globular protein body with four distinguishable functional surfaces: plus end, minus end, H3 surface, and ML surface (Figure 1) [19,21].

The plus and minus ends of adjacent tubulins bind to each other, participating in longitudinal tubulin interactions necessary for the formation of both heterodimers and protofilaments. The minus end surface includes the amino acids of helix H10 and loops H3–S4, T7, and H10–S9 (Figure 1). The plus end is composed of residues from helices H1, H6, and H11; strand S3; and loops T3, T5, H6–H7, and H11–H12 (Figure 1). Therefore, the plus end surface also includes the nucleotide-binding pocket (NBP) where GTP/GDP is tethered (Figure 1). Importantly, the α-tubulin plus end is tightly associated with the minus end of the β-tubulin within the heterodimer and, thus, its NBP is permanently associated with GTP (so-called non-exchangeable or N-site). Its precise function is unknown, although mutations that alter the GTP-binding site on α can be highly deleterious [22]. By contrast, the β-tubulin plus end, including NBP, is exposed, allowing exchange of GTP to GDP (so-called exchangeable or E-site). The GTP-bound dimer acquires a so-called curved conformation, which allows it to be accommodated at the microtubule tip. In fact, cryo-EM studies show that the microtubule tip has a slightly “open” morphology (see Figure 1) (reviewed in [18,23]). After being incorporated into a microtubule wall, a heterodimer acquires a so-called straight conformation, which requires “compression” of the α-tubulin intermediate domain by moving S8 and S9 closer to H8 [24].

The hydrolysis of α-tubulin–GTP causes compaction of the heterodimer and sub-nanometer shortening of the protofilament, changing longitudinal but not lateral forces within the microtubule [25]. Although GTP-to-GDP conversion is an important factor that regulates microtubule dynamics by triggering conformational changes in tubulin dimer, several studies suggest that other tubulin regions, including intermediate and C-terminal domains, could also be involved in this process [26,27,28].

The H3 and ML surfaces are involved in lateral interactions between heterodimers of neighboring protofilaments, leading to the arrangement of the protofilments into a cylindrical structure [19]. The H3 surface consist of helix H3 and strand S3 as well as loops H1–B1, H2–B2, and H4–S4, while the ML surface contains helices H6, H9, and H10 and loops H6–H7, S7–H9 (M-loop), and H9–S8 (Figure 1) [19].

The secondary structures of the C-terminal domain, helices H11–H12, are organized into the so-called outside of the tubulin, which in the MT, is exposed to the cytoplasm. The two helices form a surface on which microtubule-associated and motor proteins can bind [19,20], while the unstructured C-terminal amino acids (10 in α-tubulin and 18 in β-tubulin) form the tubulin tail. This region is particularly variable, showing the most amino acid sequence differences between tubulin isotypes (Table 1). Such sequence divergence may provide the structural basis for the construction of specific types of microtubules and determination of their particular properties and functions [19,20].

## 3. Intrinsic Factors Affecting Microtubule Dynamics

### 3.1. Tubulin Isotypes and Microtubule Dynamics

The human genome encodes seven α- and nine β-tubulin isotypes [29]. As a consequence, MTs composed of mixed combinations of α- and β-tubulin isotypes show tissue and developmental stage specificity and different properties in different cell types [30,31].

Human α-tubulins belongs to four classes (out of nine characterized in mammals; see [32,33]): αI (isotypes αIa and αIb), αIII, αIV (isotypes αIVa and αIVb), and αVIII (Table 1). Interestingly, two classes show tissue-specific distribution, with αIII restricted mainly to testis and αVIII to heart and skeletal muscle, while the other two are widely distributed [34,35,36,37,38,39]. Although the tissue or cell type-specific functions of α-tubulin isotypes were only recently addressed for several isotypes (αIa in neuron migration [40], αIVa in platelets [41], and αVIII in brain and testis [42]), the role of α-tubulin isotypes in MT dynamics remains unknown.

Human β-tubulins belong to seven classes (out of nine in mammals): I–VI and VIII (Table 1). From published data and the publicly available National Center for Biotechnology Information (NCBI) database, it seems that the most ubiquitously expressed isotypes are βI, βIVb, βV, and βVIII, while other β-tubulins have more specific tissue distribution (Table 1) [43]. Interestingly, although they are widely distributed, βI, βV, and βVIII were shown to have nonredundant roles in specific cellular or developmental processes. The silencing of βI expression in differentiating human neuroblastoma cells was lethal, while depletion of βI in undifferentiated cells had no apparent effect on cell survival [44]. On the other hand, βVIII is essential for oocyte maturation and early embryo development [45].

βII, βIII, and βIVa are mainly expressed in the brain (Table 1) [43,46,47]. Accordingly, experimental data indicate their specific role in processes related to the nervous system. The silencing of βII inhibits neurite outgrowth in differentiating neuroblastoma cells, while βIII was suggested to protect cells against oxidative stress [44]. However, more recent data with the use of *Tubb3* knockout mice indicate that the βIII isotype functions in the process of axon growth and nerve regeneration [48]. An interesting observation was made for βIVa: mutations in this tubulin can cause dysfunction of neurons, oligodendrocytes, or both [49].

Not much is known about the functions of other β-tubulin isotypes, βIVb and βVI. The latter shows slightly higher expression in bone marrow (Table 1) and is essential for the formation of platelet cytoskeleton (reviewed in [50]); in many publications, the *TUBB1* gene product, following protein names in the database, is called β1 or β-1 instead of βVI).

How do particular α - and β-tubulin isotypes relate to MT dynamics? The answer to this question came from studies focusing mainly on the comparison of the properties of three neuronal isotypes, βII, βIII, and βIV. The molecular surfaces of tubulins are very similar, considering the level of amino acid similarity, which for α-tubulins reaches approximately 90–95%, and is slightly lower for β-tubulins at ~85–95% (with βVI being the most divergent, with approximately 75% similarity to other isotypes). Most amino acid substitutions are accumulated within the C-terminal tubulin tail (Table 1), suggesting that this part could be responsible for the potential variation of properties of tubulin isotypes and, subsequently, the differences in MT dynamics [29]. However, several β isotypes also have unique substitutions in other regions important for the formation of functional surfaces and pockets.

**Table 1:** Features of human tubulin isotypes.

Tubulin Isotype	Gene Name	Expression	Cell Type-Specific Functions	Sequence of C-Terminus *	Reference
αIa	*TUBA1A*	ubiquitous	neuron migration	VEGEGEEEGEEY	[40,51]
αIb	*TUBA1B*	ubiquitous		VEGEGEEEGEEY	[52]
αIc	*TUBA1C*	ubiquitous		ADGEDEGEEY	[53]
αIIIc	*TUBA3C*	testis		VEAEAEEGEEY	[54]
αIIIe	*TUBA3E*	testis		VEAEAEEGEAY	[55]
αIVa	*TUBA4A*	ubiquitous	platelet biogenesis	YEDEDEGEE	[41,56]
αVIII	*TUBA8*	high: heart and skeletal muscle moderate: brain, testis, and thyroid very low: all other tissues	spermatogenesis	FEEENEGEEF	[35,42,57]
βI	*TUBB*	ubiquitous	survival of differentiated neuroblastoma	EEEEDFGEEAEEEA	[43,44,58]
βIIa	*TUBB2A*	high: brain very low: all other tissues	neurite outgrowth	DEQGEFEEEEGEDEA	[43,44,59]
βIIb	*TUBB2B*	high: brain very low: all other tissues	neurite outgrowth	DEQGEFEEEEGEDEA	[43,44,60]
βIII	*TUBB3*	moderate: brain low: testis	oxidative stress axon and nerve regeneration	EEEGEMYEDDEEEESEAQGPK	[43,44,48,61]
βIVa	*TUBB4A*	high: brain moderate/low: other tissues	neurons and oligodendrocyte function	EEGEFEEEAEEEVA	[43,49,62]
βIVb	*TUBB4B/* *TUBB2C*	ubiquitously expressed high: testis, bone marrow, and heart moderate/low: other tissues		EEEGEFEEEAEEEVA	[43,63]
βV	*TUBB6*	ubiquitous at low levels	related to skeletal muscle regeneration	NDGEEAFEDEEEEIDG	[43,64,65]
βVI	*TUBB1*	very low level in all tissues, highest in bone marrow and spleen	platelet cytoskeleton	VLEEDEEVTEEAEMEPEDKGH	[43,66]
βVIII	*TUBB8*	very low in all tissues, highest in testis	oocyte maturation, early development	EEEEDEEYAEEEEVA	[67]

Blue: negatively charged amino acids, red: positively charged amino acids, yellow: C-terminal tyrosine residue, purple: phosphorylatable residue, * sequences were taken from the NCBI database.

Among the investigated β-tubulin isotypes (βII, βIII, and βIV), βIII is the most divergent, with substitutions in globular protein body including: (i) helix H3 (serine in positions 124 and 126 is substituted by cysteine and asparagine, respectively), (ii) loops H1–S2 and H2–S3 (structures involved in the formation of H3 surface), (iii) structures located proximal to the ML surface (H6–H7 loop threonine 217 and S7–H9 loop serine 275 substituted to alanine) [68], and (iv) T7 loop (minus end surface) near colchicine-binding site (cysteine 239 substituted by serine). Moreover, the C-terminal tail of βIII is very divergent. It contains positively charged lysine (Table 1) and a phosphorylatable serine [69,70]. These variations are potentially important for the overall tubulin structure; in fact, an atomic model of βIII-containing MTs shows slight but significant displacement of the H1–S2 loop and part of the ML surface-forming structures with respect to βII-containing MTs [68]. However, how βIII substitution relates to its specific properties is mainly unknown.

For more than two decades, it has been known that MTs assembled from βIII tubulin are more dynamic than MTs containing βII or βIV [9]. More recent data indicate that the main difference is caused by the significantly increased catastrophe rate (depolymerization) of βIII-containing MTs [27,71,72], while the growth rate seems to be similar [27,71] or only slightly lower [72]. The dynamic features of MTs assembled with specific isotypes were retained when the C-terminal tails of βII and βIII were interchanged, indicating that the dynamic properties of these two tubulins are “encoded” within the main globular protein body [71]. Remarkably, not only dynamics, but also resistance to depolymerizing factors and structural features vary between βII- and βIII-containing MTs, with the former showing lower resistance to depolymerizing agents and more protofilaments in the MT wall (14) than the latter (βIII contains 13 protofilaments and shows more resistance to depolymerizing factors) [68]. Interestingly, when two populations of tubulin heterodimers are mixed with different stoichiometry, assembled MTs show intermediate dynamics [68,71,72].

On the other hand, at low tubulin concentration, the MTs nucleate much more slowly if the tubulin heterodimers contain βIII-tubulin compared to βII or βIV. Interestingly, this difference can be abolished by the proteolytic removal of C-terminal tail [8]. This result indicates that although it is not crucial for dynamic tubulin properties, the tubulin C-terminus can influence other features that could be important for MT cytoskeleton formation within the cell.

To summarize, growing evidence indicates that the intrinsic properties of tubulin isotypes, the expression of specific isotypes, and their ratio within the cell are significant factors influencing MT dynamics.

### 3.2. Post-Translational Modifications of Microtubules

Tubulin modification sites, modifying enzymes, and functions of post-translational modifications (PTMs), including the impact on MT dynamics, have recently been broadly reviewed [12,33,73,74]. Thus, here, we will only briefly summarize how PTMs affect MT dynamics.

Both α- and β-tubulin undergo a number of post-translational modifications that change the properties of the free tubulin heterodimers and microtubules. Tubulin PTMs can modulate MT dynamics directly or indirectly by influencing the interactions between MTs and microtubule-interacting proteins (which can stabilize, destabilize, or cut microtubules).

While for the vast majority of modifying enzymes, a tubulin heterodimer already incorporated into the microtubule lattice is a preferred substrate, in some cases, free tubulin heterodimers are effectively modified. The latter can affect the binding of tubulin heterodimers to the microtubule plus end and, thus, affect microtubule growth and stability. For instance, acetylation of lysine 252 of β-tubulin by San acetyltransferase slows down the rate of tubulin incorporation into the microtubule and consequently reduces the rate of MT assembly [75]. Phosphorylation of serine 172 of β-tubulin by minikinase/DYRK1a (neurons) or cyclin-dependent kinase Cdk1 (mammalian mitotic cells) inhibits the incorporation of tubulin heterodimers [76,77], while heterodimers containing αIc-tubulin phosphorylated at serine 165 assemble more effectively than unmodified ones [78,79]. Serine and threonine residues of α- and β-tubulin can also be modified by *O*-Glc-NAcylation [80,81] and, in vitro, such modified tubulins are not incorporated into microtubules [81].

The presence of differentially modified MTs within the cell is crucial in the assembly, disassembly, and rearrangement of the microtubular cytoskeleton [82]. Newly polymerized dynamic microtubules are highly tyrosinated (besided αIVa, tyrosine is encoded in the C-terminus of α-tubulins, see Table 1). With time, the tyrosine is removed by the vasohibin family, VASH1 and VASH2 tubulin detyrosinases, generating so-called detyrosinated tubulin. Detyrosinated tubulin is found in spindle and is essential for correct chromosome congression [83]. Glutamic acid residue, which is the most frequent penultimate residue in α-tubulin, can also be removed by cytosolic carboxypeptidases, irreversibly forming so-called Δ2-tubulin [84,85,86,87]. After depolymerization, free detyrosinated α-tubulin can be re-tyrosinated by tubulin tyrosine ligase (TTL) [88,89].

The α-tubulins I, II, III, IV, and VII have a lysine at position 40. Acetylation of lysine 40 residue by α-tubulin N-acetyltransferase 1 (ATAT1) has been known to mark stable microtubules [90,91,92,93]. The modification is reversible, and tubulin deacetylation is carried out by histone deacetylase 6 (HDAC6) [94] and Sirt2 deacetylase [95]. Recent analysis using high-resolution cryo-electron microscopy showed that the acetylation of lysine 40 restricts the range of motion of the loop containing K40, likely weakening the lateral contacts between protofilaments [96] and thus increasing MT flexibility [97,98].

Polyamination of α- and β-tubulin by a transglutaminase causes the formation of hyperstable, cold-resistant microtubules. This modification is important for neuronal development and axon maturation [99]. The positions of the main polyamination sites near the GTP pocket (glutamine 15 in β-tubulin) and α-tubulin minus end suggest that tubulin polyamination could affect GTP binding or hydrolysis and microtubule lattice stabilization [99].

Glycylation and glutamylation of α- and β-tubulin can occur as mono- or polymodification, and glycyl or glutamyl residues are ligated to the glutamic acid residues within the C-terminal tail [73,92]. These tubulin modifications are catalyzed by enzymes related to TTL, called tubulin tyrosine ligase-like (TTLL) [73]. The reverse reaction (deglutamylation) is carried out by cytoplasmic carboxypeptidases (CCPs). To date, the identity of tubulin deglycylase remains unknown [92].

### 3.3. Microtubule-Associated Proteins and Microtubule Dynamics

Microtubule-associated proteins (MAPs) are another intrinsic factor affecting microtubule dynamics (reviewed in [100,101,102]). Generally, MAPs function as MT stabilizers or destabilizers; however, stabilization/destabilization of MTs can be achieved by affecting one of several processes, including MT nucleation [1] and stabilization/destabilization of the MT ends [2] or of the MT lattice [102,103]. For this reason, MAPs are divided into several functional categories: (i) microtubule nucleators, (ii) MT end-binding proteins, (iii) lattice-binding proteins also known as structural MAPs, (iv) enzymes severing or depolymerizing microtubules, and (v) motor MAPs (kinesin, dynein) that generate forces and use microtubules as tracks for intracellular transport [102]. Intriguingly, some MAPs can participate in several MT dynamics-related processes. For example, XMAP215 can be classified as both an MT nucleator and a plus end-binding protein [1,2] while kinesin-13 family proteins are motor proteins that bind to microtubule plus ends and have MT-depolymerizing properties [104,105]. Because MAPs form a large class of proteins and a number of high-quality reviews on this topic are already available, we will only provide a short overview of these proteins, highlighting the relationship between MAPs and MT dynamics.

Microtubule nucleators enhance the initiation of MT formation from both γ-TuRCs and microtubule seeds and stabilize growing MTs. This category of MAPs includes XMAP215/CKAP5, TPX2 (targeting factor for Xklp2), DCX (doublecortin), CAMSAP (calmodulin-regulated spectrin-associated protein)/Patronin, CLASP (cytoplasmic linker associated protein), and p150Glued proteins [1,23]. They are believed to act mainly as enhancers of longitudinal and lateral contacts between tubulin dimers [1]. Interestingly, TPX2, besides its nucleating activity, also suppresses MT depolymerization and shrinkage, and thus increases MT stability [106].

Proteins that bind to MT ends are specific to either the plus (so-called +TIPs [107]) or minus end (so-called −TIPs [108]). Plus TIPs belong to approximately 20 different families of proteins [107]. EB (end-binding) proteins form a core of +TIP network and the majority of studies suggest that they stabilize or protect the MT plus end [107]. EB proteins interact with both the MT plus end and other +TIP proteins that can be either stabilizers (as CLIP-170 (cytoplasmic linker protein 170)/CLIP1 and CLASP proteins), destabilizers (as kinesin-13 family proteins), or polymerases (as XMAP215 protein). Within the cell, the interplay between these proteins results in MT growth or shrinkage (reviewed in [2,23,107]).

Stathmin-1, also known as Op18 (oncoprotein 18), binds to the MT plus end but is not included in the +TIP protein class. It was first discovered as an oncoprotein highly expressed in some types of leukemia, breast, and ovarian cancers [109]. Stathmin-1 causes a decrease of the MT polymer mass by two mechanisms: (i) by binding two tubulin dimers in a curved conformation and inhibition of their incorporation into microtubule and (ii) by interfering with the lateral bonding between tubulin subunits, leading to destabilization of the microtubule tip and MT shrinkage [110,111].

A recently described class of −TIP, includes CAMSAP proteins and the KANSL complex [108]. In mammals, CAMSAP proteins protect the MT minus end against the depolymerizing activity of kinesin-13. Additionally, CAMSAP2 and 3 proteins decrease the rate of tubulin incorporation at the minus end, decreasing its dynamics [112]. The functions of KANSL complex are still unknown [108].

The lattice-binding MAPs include classical MAPs, MAP1, MAP2, MAP4, MAP6, MAP7, and Tau, which promote polymerization, stabilization, and bundling of microtubules (reviewed in [102]). They also regulate the association of MTs with other cytoskeletal fibers, organelles, and membranes, and influence the ratio of transport along MTs and MT severing by physically blocking the access of motors and severing enzymes. Additionally, structural MAPs can regulate the number of MT protofilaments [102]. Interestingly, it seems that MAP6 has unique properties and functions as it is a microtubule luminal protein and protects MTs against drug and cold-dependent destabilization [113]. With the exception of MAP4, which is a ubiquitous protein, and the expression of MAP7 in epithelial cells, the expression of structural MAPs is mainly restricted to the brain [114].

Microtubule organization is also regulated through the microtubule-severing proteins katanin, spastin, and fidgetin, whose activity can lead to MT shortening or even depolymerization, but also to the formation of numerous microtubule seeds that serve as MT nucleation templates and free tubulin dimers that can be incorporated into new microtubules. Therefore, severing activity can have both a negative and positive effect on MT dynamics and microtubule polymer mass (reviewed in [103]).

It should be noted that, within a cell, the organization and dynamics of MTs is a result of the interplay between tubulin isotypes, their posttranslational modifications, and microtubule-associated proteins. For example, tyrosination increases the affinity of MT to the stabilizing protein, CLIP-170/CLIP-1 but, also to depolymerizing proteins from the kinesin-13 family [115,116,117]. Similarly, tubulin polyglutamylation affects the interactions of MT with several MAPs, including Tau, MAP1, and MAP2, but also with MT-severing proteins [118,119,120].

## 4. Extrinsic Factors Affecting Microtubule Dynamics

### 4.1. Microtubule-Targeting Agents

The surface of the globular part of tubulins contains several pockets that can be intercalation sites for MTAs. These compounds, while embedded in the tubulin structure, can alter the microtubule dynamics. This feature of MTAs is used in cancer therapy, as it was shown that treatment of cancer cells with MTAs led to the mitotic arrest and consequent cell death [18,121]. Many MTA compounds are produced by plants, fungi, and invertebrates as a natural defense against antagonists, competitors, or parasites (for a review, see [122]).

**Figure 2:**
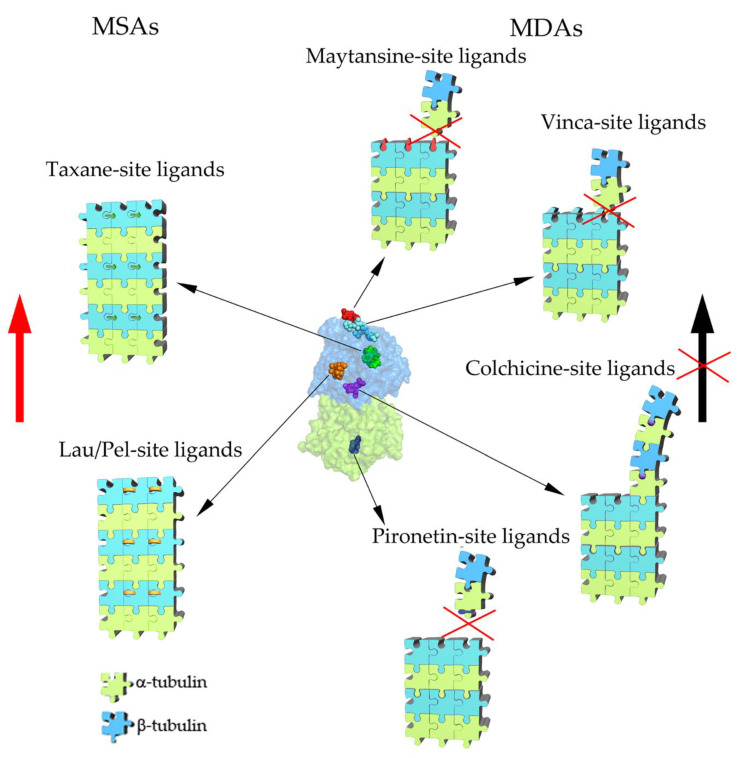
Location of the microtubule-targeting agent (MTA)-binding site on tubulin and the MTA mechanism of action. Microtubule-stabilizing agents (MSAs) (taxane and laulimalide/peloruside-site ligands) promote microtubule (MT) growth (red arrow) by stabilizing lateral contacts between neighboring heterodimers (see text). MDAs inhibit MT growth and destabilize MTs (crossed out black arrow) by inhibiting addition of new heterodimers at the MT plus end (maytansine- and pironetin-site ligands) or by inhibiting transition of heterodimer structure to straight conformation (colchicine- and vinca-site ligands) (see text).

Currently, six MTA-binding sites, named after the main compounds with affinity to them, have beem described (Figure 2). Four pockets are located on β-tubulin: taxane, laulimalide/peloruside, vinca, and maytansine sites. The colchicine site is islocated near the intradimer interface between the α- and β-tubulin subunits, while the pironetin site is a binding pocket located on the α-tubulin surface (for a review, see [18]). Taxane and laulimalide/peloruside sites bind compounds that stabilize microtubules (MSAs), while the other four pockets accommodate factors that destabilize MTs (MDAs).

### 4.2. Tubulin Pockets

#### 4.2.1. Taxane Site

Paclitaxel, a tetracyclic diterpenoid originally isolated from *Taxus brevifolia* in the 1960s [123], was approved for the treatment of ovarian cancer in 1992 by the US Food and Drug Administration (FDA) as Taxol^®^. Now, paclitaxel is produced by a semi-synthetic route by modifying 10-deacetylbaccatins III derived from the European yew *Taxus baccata* [124].

**Figure 3:**
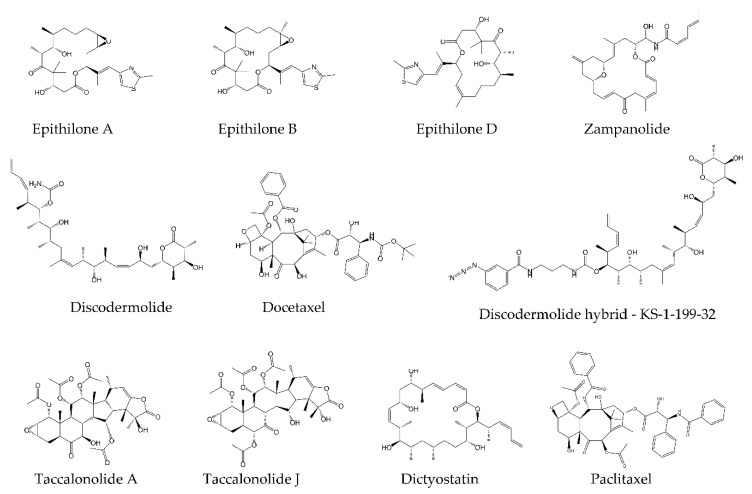
Structure of docetaxel; epothilones A, B, and D; discodermolide (DDM), KS-1-199-32; dictyostatin; taccalonolide A and J; zampanolide; and paclitaxel.

Paclitaxel and its derivates are used in diverse cancer therapies and are characterized by high neurotoxicity, myelosuppression, poor water solubility, and the occurrence of multidrug resistance (MDR) in treated tumors (see below). This led to the search for and discovery of other compounds that enhance microtubule stabilization, including epothilones A, B, and D; discodermolide (DDM) and the DDM–paclitaxel hybrid KS-1-199-32; dictyostatin; taccalonolide A and J; and zampanolide (Figure 3) (for reviews, see [18,125,126]).

Epothilones A and B are macrolide drugs (natural products that consist of a large macrocyclic lactone ring) produced by the myxobacterium *Sorangium cellulosum* [127]. Unlike paclitaxel, they are highly soluble in water and are not a substrate for P-glycoprotein, which actively transports drugs out of the cell. A semi-synthetic derivate of epothilone B, ixabepilone, was approved for cancer treatment [128,129].

Zampanolide, a sponge-derived macrolide, and taccalonolide A and J, polycyclic steroids isolated from plants of the genus *Tacca*, were more recently discovered and are still under investigation, but are promising antitumor drug candidates [130]. They both have the unique ability to bind covalently to taxane-site residues asparagine 228/histidine 229 and aspartic acid 226 [130,131].

Taxane-site targets are used in the treatment of various cancers, including ovarian (paclitaxel), breast (paclitaxel, docetaxel, larotaxel, ixabepilone), lung (docetaxel, epothilone B, larotaxel), bladder (larotaxel), hormone-resistant prostate cancer (cabazitaxel), and others [128,129,132,133,134,135,136,137].

The taxane (Figure 2) site is located near the ML surface, on the “inside” side of the tubulin (which in MT, faces the lumen), and is formed by hydrophobic residues of H7, S7, and loops H6–H7, S7–H9 (M loop), and S9–S10 (Figure 2) [19,20,138]. All compounds (Figure 3) that bind to the taxane site form hydrophobic and polar contacts with pocket amino acids and strengthen the lateral contacts between heterodimers of adjacent protofilaments, leading to MT stabilization (Figure 2). The mechanism of microtubule stabilization is compound-specific [25,131,138,139]. Several compounds that contain side chains, such as epothilone A and zampanolide, engage with the M loop, stabilizing it into a short helix [138]. Significantly, a similar helical conformation of the M loop was observed in native polymerized MTs [25,138], indicating its importance in the formation of stable MT. Other compounds (e.g., taccalonolide A and J) cause displacement of the M-loop into a more open conformation, facilitating lateral interactions between adjacent protofilaments [131]. By contrast, paclitaxel was suggested to stabilize MTs via an allosteric mechanism by preventing dimer compaction after GTP hydrolysis [25,138]. A similar indirect effect was also proposed as an additional mechanism of MT stabilization for taccalonolide AJ and zampanolide [130,131].

#### 4.2.2. Laulimalide/Peloruside Site

**Figure 4:**
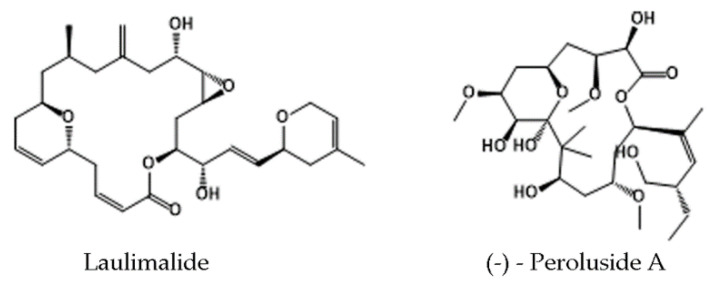
Structure of laulimalide and peloruside A.

Laulimalide and peloruside A (Figure 4), macrolides originally isolated from marine sponges (*Cacospongia mycofijiensis* and *Mycale hentscheli*, respectively) [140,141,142], are promising non-taxane-site MSAs. Although high-resolution X-ray crystallography and cryo-EM have resolved the tubulin–compound interactions in detail [138,143], biological investigation of these MTAs is still ongoing [144,145,146].

The laulimalide/peloruside site is positioned at the opposite side of the ML surface with respect to the taxane site, i.e., the “outside” surface of the MT wall (Figure 2), and is formed by hydrophobic and polar residues of H9 (including a short loop that divides H9 into H9 and H9’), H10, and loop H10–S9 of β-tubulin (Figure 2) [138,143].

After binding laulimalide or peloruside to the β-tubulin pocket, MTs are stabilized by two main mechanisms which strengthen the lateral contacts between neighboring protofilaments. First, the β-tubulin M loop shifts to an “open” conformation (without forming the regular secondary structure). Second, the position of both agents within the pocket allows their interaction with the H3 surface of the adjacent heterodimer, which leads to bridging of neighboring protofilaments (Figure 2) [143]. In the case of peloruside A, an especially strong effect is observed in the seam, where the lateral contacts are weaker. It was also proposed that both compounds fix structures located near the M loop and, thus, have an additional allosteric effect on MT stabilization [143].

#### 4.2.3. Vinca Site

**Figure 5:**
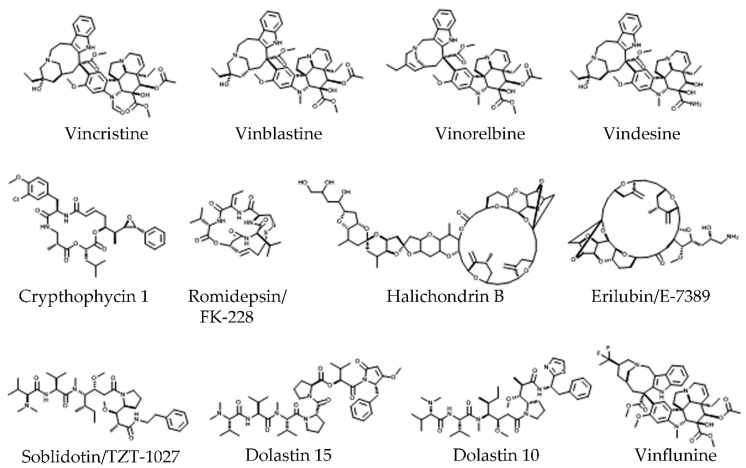
Structure of vincristine, vinblastine, vindesine, vinorelbine, vinflunine, dolastatin-10, dolastatin-15, TZT-1027, cryptophycin 1, FK228, halichondrin B, and E-7389.

The naturally occurring vinca alkaloids (vincristine and vinblastine) were discovered in periwinkle (*Catharantus roseus* G. Don.) in the late 1950s (Figure 5). These are first-generation vinca alkaloids that have achieved significant clinical progress [147,148]. The therapeutic success of vinca alkaloids in the treatment of hematological cancers (mainly childhood leukemia) [149] led to the development of diverse semi-synthetic analogs (Figure 5) [150], including vindesine, vinorelbine, and vinflunine, the latter used for the treatment of solid tumors, particularly metastatic breast cancer [151]. However, similar to taxanes, vinca alkaloids have severe side effects (peripheral neuropathies and reversible myelosuppression) [152,153].

Besides vinca alkaloids, several other groups of compounds were also shown to target the vinca site, including peptides, depsipeptides, and macrolides, and some have been used in clinical trials (reviewed in [18,125,154,155]). Currently, vincristine and vinblastine are used for the treatment of breast cancer, lymphomas, and sarcomas [150], vinorelbine for breast and lung cancer, sarcomas, and glioma [150,156], vindesine for lung cancer [150], vinflunine for urothelial cancer [157,158], vintafolide (vinflunine and folate) for lung, ovarian, and endometrial cancer [159], eribulin for liposarcomas, bladder cancer, and metastatic breast cancer [160,161], and dolastatin 10 for solid tumors [162].

The vinca site is located at the plus end surface of β-tubulin and is formed by residues of H6 and loops T5 and H6–H7; however, several agents also bind to H7 and β-tubulin-bound nucleotide sites [163,164,165]. The vinca-site ligands also form connections with α-tubulin of the subsequent dimer, interacting with its minus end surface structures, including H10, S9, and T7 loop [163].

The binding of ligands to the vinca site alter the surface of the β-tubulin plus end, forming a so-called wedge (Figure 2) [163], thus interfering with the incorporation of new heterodimers at the MT plus end. As a result, the plus end heterodimers remain in curved conformation, which inhibits formation of the MT wall and leads to destabilization [163]. It was also shown that vinca-site ligands can cause the formation of ring-like tubulin oligomers, decreasing the level of free tubulin available for polymerization (Figure 2) [163,166]. Additionally, several vinca-site compounds were shown to have an allosteric effect on the inhibition of lateral contacts between dimers by stabilizing the M loop in the interaction-incompetent conformation [165].

#### 4.2.4. Maytansine Site

**Figure 6:**
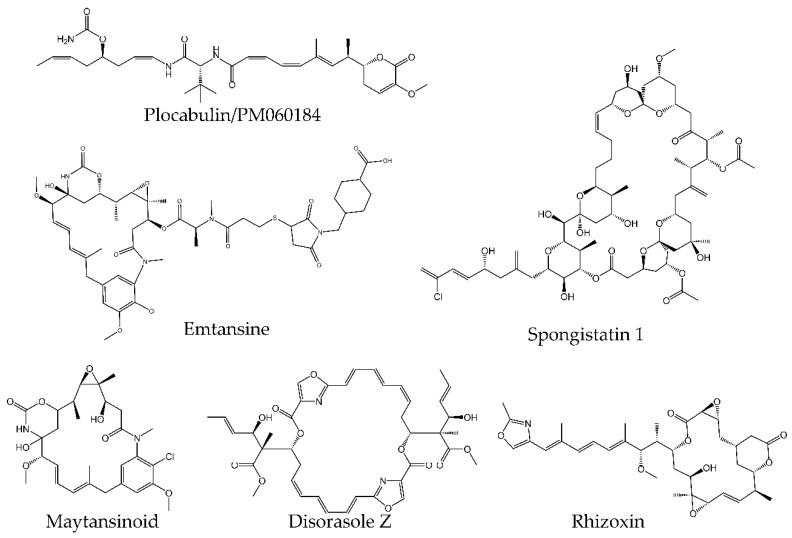
Structure of maytansinoid, disorazole Z, emtasine, rhizoxin, PM060184, and spongistatin 1.

Maytansine and its derivatives were first isolated from an African shrub, *Maytenus ovatus* [167]. They belong to the natural product group of maytansinoids, macrolides of the ansamycin type (Figure 6). Later, other groups of compounds, including macrocyclic polyketides (disorazole Z, Figure 6), macrocyclic lactones (rhizoxin, Figure 6), lactones (plocabulin, PM060184, Figure 6), and macrocyclic lactone polyethers (spongistatin 1, Figure 6) were isolated.

It is worth noting that PM060184 is currently under clinical evaluation [168], while ado-trastuzumab emtansine was recently approved for adjuvant treatment of patients with HER2-positive early breast cancer [169] and was shown to prolong patient survival with a manageable safety profile [170].

The maytansine site is located in close vicinity to NBP and the vinca site, but is formed by other structures, including H3’, H11, H11’, and loops H11–H11’, T3, and T5 (Figure 2) [171].

The experimental evidence indicates that the inhibitory effect of maytansine-site ligands is a direct consequence of the occupation of the β-tubulin pocket. In growing microtubules, the maytansine-binding pocket of the MT plus end β-tubulin accommodates the minus end structures of the α-tubulin of a newly added heterodimer, including S8, H8, and loop H10–S9 [171]. Incorporation of maytansine-site ligands prevent this interaction, impeding MT elongation (Figure 2).

#### 4.2.5. Colchicine Site

**Figure 7:**
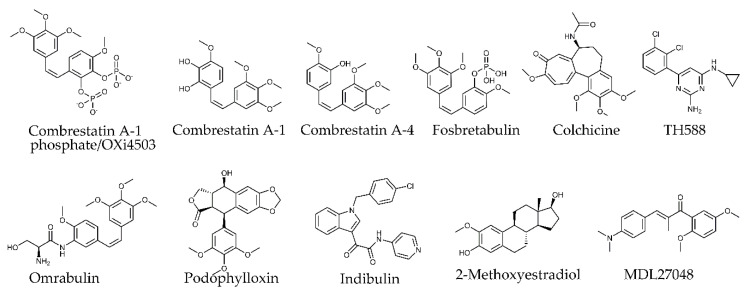
Structure of colchicine, combretastatin A-1, combretastatin A-4, OXi 4503, fosbretabulin, ombrabulin, 2-methoxyestradiol, chalcone: trans-1-(2,5-dimethoxy)-3-[4 (dimethylamino) phenyl]-2-methyl-2-propen-1-one (MDL 27048), podophyllotoxin, indibulin, and TH588.

Colchicine (Figure 7) was isolated from autumn crocus *Colchicum autumnale.* This alkaloid contains three rings, of which rings A and C bind to β-tubulin, while aromatic ring B binds to α-tubulin [172]. Colchicine–tubulin binding is a slow, strongly temperature-dependent, and practically irreversible process [173]. Although colchicine has been used clinically in the treatment of nonneoplastic diseases (gout, familial Mediterranean fever), neither colchicine nor other related compounds were successful as chemotherapeutic agents owing to their severe toxicity to normal tissues at doses required for antitumor effects [174,175].

Over the last few decades, compounds of low toxicity which target the colchicine site have been reported (Figure 7), including derivatives of stilbenoid–combretastatins (combretastatin A-1 phosphate/OXi4503, combretastatin A-1, combretastatin A-4, fosbretabulin, ombrabulin), chalcones (MDL 27048), compounds with furonaphthodioxole skeleton (podophyllotoxin), derivatives of indole (indibulin), and natural metabolite of estradiol (2-methoxyestradiol). While no colchicine-site MTAs are currently approved for cancer treatment, several are in phase I/III clinical trials [174] (for a review, see [175]).

Recently, a compound initially designed as MTH1 (Mut T homolog 1) inhibitor, TH588, was shown to dock into the colchicine-binding pocket [176]. By reducing microtubule plus end dynamics, this cyclopropyl analog affects tubulin polymerization, resulting in disruption of mitotic spindles, prolongation of mitosis and, eventually, apoptosis [176,177,178]. Preclinical studies show promising results for the use of TH588 as an anticancer drug [179,180].

The colchicine-binding site is located near the plus end surface of β-tubulin in the center of the tubulin heterodimer at the interface between α- and β-tubulin. It is a big pocket formed by the hydrophobic and polar residues of H7, H8, S7, S8, and loop H7–H8 (T7 loop) that can be divided into three zones: central zone 2 and two accessory zones, zone 1 facing α-tubulin and zone 3 buried deeper within the β-tubulin pocket [181,182].

Binding of colchicine-site ligands to heterodimer causes its stabilization in the curved conformation (Figure 2) [181]. As mentioned, during MT polymerization, tubulin dimers at the MT tip undergo a transition from curved to straight conformation, which requires a shift of several β-tubulin structures (S8–S9 and H8) closer to each other. As a result, the colchicine pocket is contracted [24,181]. While the colchicine pocket is occupied by a ligand, such conformational changes cannot occur, making colchicine ligand-bound heterodimer incompetent for polymerization [24,181].

#### 4.2.6. Pironetin Site

**Figure 8:**
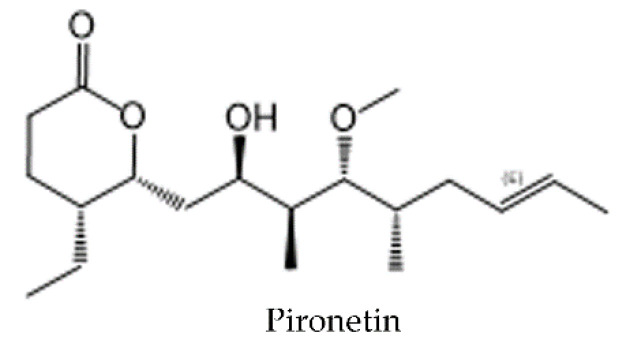
Structure of pironetin.

Pironetin (Figure 8), a polyketide, is a natural product that was first extracted from fermentation broths of *Streptomyces* sp. [183,184]. It is worth noting that pironetin is, to date, the only known compound that exclusively targets the α-tubulin subunit and covalently binds to Cys316 of α-tubulin [185,186]. The molecule and its derivates are currently under investigation and display promising anticancer properties (reviewed in [187]).

The pironetin-binding site is the only known pocket on α-tubulin targeted by MTAs. Its main part is formed by residues of S8, S10, and H7, but residues of S4, S5, and S6 also participate in the ligand accommodation [185]. The binding of pironetin leads to conformational changes of α-tubulin within the minus end, including disordering of loop H7–H8 (T7 loop) and part of H8 [185]. Since these structures are required for the formation of longitudinal interactions within protofilaments, it was proposed that pironetin prevents MT polymerization by the formation of assembly-incompetent pironetin-bound tubulin dimers (Figure 2) [185].

## 5. Factors Affecting Microtubule Dynamics in Cancer Cells

Carcinogenesis is a multistep process involving, among other actions, a remodeling of the cytoskeleton. The transformation from highly polarized epithelial cells to multipolar spindle-like metastatic cells that are able to detach from the extracellular matrix and migrate requires extensive reorganization of the cytoskeleton, including microtubules, during a process called epithelial–mesenchymal transition (EMT) (for a review, see [188]). The abnormalities of mitotic spindle and consequent aberrant cell cycle progression and division lead to genomic instability (for a review, see [189]). Thus, it is not surprising that numerous alterations in tubulins, including mutations and variations in isotype expression level, post-translational modifications, and MAP composition, were identified in cancer cells.

### 5.1. Tubulin Isotypes in Cancer and Anticancer Drug Resistance

Altered expression of tubulin isotypes is considered to be a hallmark in a range of cancers. Analysis of clinical specimens has shown that in many cancers, a high expression of several β-tubulin isotypes correlates with aggressive clinical behavior, chemotherapy drug resistance, and poor patient outcome [121]. Strikingly, little is known about the level of tubulin isoforms in primary nontreated cancers, while numerous studies indicate their variations after chemotherapy, especially after taxane-based treatment [121]. In fact, some data show that in cancer cell lines, paclitaxel can itself induce the expression of specific tubulin isoforms [190].

An increase in βI expression was observed in several cancers, including breast, colon, and kidney cancer, while its level was decreased in prostate cancer (Table 2) [43,191,192,193]. In the case of ovarian cancer, the data are inconsistent as both increased and invariant levels of βI expression were reported [43,191,194,195]. High βI expression is associated with the acquisition of chemoresistance to MTAs and poor prognosis in ovarian serous carcinoma and lung adenocarcinoma, but not in lung squamous cell carcinoma [191,193,196]. Interestingly, recent data show that experimentally lowering βI level by siRNA or mir-195 microRNA sensitizes adenocarcinoma cell lines to paclitaxel and eribuline [193], indicating a direct correlation between βI level and MTA resistance, at least in non-small-cell lung adenocarcinomas.

βIIa and b differ by only two amino acids and, thus, the expression of these two isotypes can be distinguished at the RNA level but not at the protein level. Using PCR, it was shown that βIIa is increased in NSCLC, prostate, and ovarian cancers, and decreased in kidney, colon, and breast cancers [43], while βIIb is increased in ovarian cancer and decreased in kidney, colon, and breast cancers [43]. Increased βIIb isoform was also recently associated with the metastatic stage of melanoma, indicating its role in EMT transition [197]. βII was also examined at the protein level in several cancer types, including head and neck carcinomas (LASCCHN), ovarian carcinoma, colorectal cancer, and breast cancer cell lines [194,196,198].

**Table 2:** Tubulin isotypes as survival and MTA-resistance prognostic markers in cancer.

High Level of Tubulin Expression	Cancer	Prognosis	Resistance to	Reference
αIa	renal	poor	n/a	[38]
αIb	hepatocellular carcinoma	poor	paclitaxel	[199]
	renal, breast		n/a	[38]
αIc	liver, renal, pancreatic, colon, breast, lung	poor	n/a	[38]
αIVa	liver	poor	n/a	[38]
βI	ovarian	not determined	paclitaxel	[191]
	breast	not determined	docetaxel	[200]
	NSCLC adenocarcinomas	poor	paclitaxel and eribulin	[193]
βII	breast	not determined	docetaxel	[196,201]
	lung adenocarcinoma cell line	not determined	paclitaxel	[191]
βIIa	urothelial	poor	n/a	[38]
	renal	good	n/a	[38]
βIIb	endometrial	poor	n/a	[38]
βIII	prostate	poor	docetaxel	[202,203]
	colon	poor	paclitaxel	[204]
	bladder, cisplatin resistant	poor after paclitaxel chemotherapy	n/a	[205]
	gastric	poor	n/a	[206]
	gastric metastatic	poor after taxane chemotherapy	n/a	[207]
	uterine serous carcinoma	poor	paclitaxel, sensitivity to epothilone	[208]
	lung carcinoma cell line	n/a	epothilone	[209]
	NSCLC	poor	vinorelbine	[210]
	NSCLC stage III/IV	poor	vinorelbine	[211]
	NSCLC stage I/II	good after cisplatin/vinorelbine adjuvant chemotherapy	n/a	[212]
	ovarian	poor	n/a	[213]
	ovarian clear cell carcinoma	good after taxane based chemotherapy		[214]
	breast	poor	n/a	[215]
	breast	not determined	sensitivity to taxanes	[216] *
	metastatic breast	invariant	sensitivity to docetaxel treatment	[217]
	melanoma	difference not statistically significant	paclitaxel	[218]
βIVa	endometrial	poor	n/a	[38]
βIVb	liver	poor	n/a	[38]
	thyroid, endometrial	good	n/a	[38]
βV	renal, urothelial	poor	n/a	[38]
	NSCLC	good	good response to paclitaxel and vinorelbine	[219]
	breast	not determined	sensitivity to taxanes	[216] *

* only mRNA level was determined.

Similar to βI, in lung adenocarcinoma, breast cancer, and breast cancer cell lines, an increased level of βII was associated with resistance to MTA [191,196,201], while in LASCCHN, it was associated with poor survival after chemotherapy [198]. By contrast, in taxane-treated ovarian carcinomas, poor outcome is associated with a low level of βII [194]. Interestingly, silencing of βII by siRNA in NSCLC adenocarcinoma and large-cell carcinoma cell lines increases cell sensitivity to vinca alkaloids but not to paclitaxel treatment [220].

In a number of cancers (mainly of epithelial origin) βII was observed within the nucleus of both cancer cells and nontransformed cells in tissues adjacent to the cancer [221]. Nuclear localization of βII was recently associated with poor outcomes in colorectal cancer patients [222].

It was surprising when the neural tubulin isoform βIII, which increases MT dynamics (see above), was discovered to be expressed in tumors with different origins. An analysis of the significant number of different types of tumors revealed that the contribution of βIII to the total tubulin pool depended on the cancer type [223]. For example, in nearly 70–80% of the examined cases of small-cell lung cancer, mesothelioma, NSCLC, adenocarcinoma and large-cell cancer, neuroendocrine pancreatic cancer, malignant melanoma, and gallbladder carcinoma, βIII was expressed at high levels [223]. By contrast, 70–95% of cases of breast cancer, colon adenoma, stomach cancer, basalioma, Warthin’s tumor, and hepatocellular carcinoma were βIII-negative [223].

In a wide range of tumors and cancer cell lines, including small-cell lung carcinoma, NSCLC, ovarian, prostate, bladder, uterine, upper gastrointestinal, colon, pancreatic, LASCCHN, and gastric cancer, βIII upregulation is associated with the development of resistance to taxane-based chemotherapy and poor clinical outcome [121,200,202,203,204,205,206,207,208,210,211,212,213,215,223,224,225,226,227,228,229,230,231,232,233]. Moreover, an increased βIII level was also shown to be associated with EMT and cell motility of colon cancer cell lines [234].

The role of the βIII isoform in tumorigenesis was confirmed in a pancreatic cell line model. Silencing of βIII expression by shRNA or mir-200c microRNA reduced cancer cell growth and tumorigenic potential both in vitro and in vivo in orthotopic and xenographic pancreatic cancer mouse models [190,230].

A high level of βIII is generally believed to be a bad prognostic marker for MTA resistance and survival. However, it was recently shown that a paclitaxel-resistant NSCLC adenocarcinoma cell line with increased βIII expression was sensitive to vinblastine and its analogs to the same extent as “parental” cells with low taxane resistance and lower βIII expression [235]. This indicates that βIII-induced taxane resistance may not influence resistance to other MTAs. In contrast to these observations, overexpression of βIII-tubulin in ovarian clear cell adenocarcinoma is a predictor of a good response to taxane-based chemotherapy, and cases with higher βIII-tubulin expression are associated with a significantly more favorable prognosis than those with lower βIII-tubulin expression [214]. A similar observation was made for early stages (I/II) of breast cancer [212].

Altered expression of the βIV isotype was also reported in numerous cancers, including ovarian, lung, prostate, breast, and kidney cancers and breast and lung cancer cell lines [43,191,194,196,220]; the data, however, are frequently inconsistent. For example, some data indicate decreased βIV in lung and breast cancer [43], while studies on cancer cell lines indicate increased βIV expression, especially in taxane-resistant cell lines [191,196].

Similar to βII, βIVb overexpression is associated with resistance to vinca alkaloids rather than taxanes. In fact, siRNA knockdown of IVb β-tubulin expression in NSCLC and pancreatic ductal carcinoma cell lines increases the response to vinca alkaloids [220,236]. Interestingly, downregulation of βIVb was recently observed in an EMT-induced colon cancer cell line and transformation of epithelium-like to spindle-like cell morphology in these cells was reversed by βIVb overexpression [237].

The level of βV expression was tested on RNA and protein levels. The level of βV RNA was shown to be reduced in most tumors (colon, ovary, prostate, breast, lung) except for kidney [43], while βV protein level was shown to be elevated in lung, breast, and ovarian cancers and decreased in prostate cancer [238,239]. In NSCLC, a low level of βV-tubulin was associated with poor prognosis after paclitaxel-based chemotherapy [219].

Based on the available data, it appears that different β-tubulin isotypes play specific roles in the cancer cell response to extrinsic factors influencing MT dynamics. For example, in breast cancer patients with both βI and βIII upregulation, response to taxane-based therapy was poor; in the group with a low level of both, the majority of patients responded well to the therapy, while in groups where one β-tubulin isotype high and another low, the response was intermediate [200]. Similar, in NSCLC patients with a low level of βV and high level of βIII expression, the outcome was much worse than in patients with high βV and low βIII, while patients with either a high or low level of both isotypes had an intermediate outcome [219].

Thus, the outcome of levels and ratios of particular β tubulin isotypes in terms of the progression of carcinogenesis appears to be specific to tumor type.

MTA resistance in cancer could also be related to mutations of β-tubulin. However, analyses of the clinical samples revealed that mutations in β-tubulin are either not present or very rare. Thus, it seems unlikely that mutations in β-tubulin could play an important role in drug resistance (reviewed in [240]).

Studies conducted on cell lines showed that mutations of predominating β-tubulin isotypes within the taxane-, colchicine-, and vinca-binding sites can be associated with altered MT dynamics and/or resistance to MTA (reviewed in [240,241,242]). Most β-tubulin mutations located within close proximity to the taxane-binding site did not change the affinity of tubulin to taxanes or epothilones, but probably destabilized MTs in the absence of any drugs [242]. Only mutation at F270V, T274I, and R282N residues were reported to have a direct effect on drug-binding affinity [242]. A similar effect was observed when point mutations were located in the M-loop (T274I, R282Q) or in helix H9, which is essential for interdimer interactions (Q292E) [242].

A recent study on a large number of samples of breast cancer tumors identified several mutations in βI, βIIa, and βIVb tubulins in which a gene-encoded residue was replaced by the amino acid present in the corresponding position in βIII [243]. It was proposed that such mutations could influence the clinical outcome in a similar manner as overexpression of βIII-tubulin [243].

### 5.2. Microtubule PTMs and Cancer

Changes in the level of tubulin modifications were linked to tumorigenesis (Table 3) [29,244]. Downregulation of TTL and increased α-tubulin detyrosination were reported during the epithelial–mesenchymal transition (EMT) that occurs during tumor invasion [245] in prostate cancer cells [246], in aggressive subtypes of breast cancer cells [247], and in primary neuroblastomas with poor prognosis [248]. The recent discovery of the vasohibin (VASH)/small vasohibin-binding protein (SVBP) complex, reported as a detyrosinating enzyme, tubulin carboxypeptidase (TCP) [249,250], provides new links between this tubulin modification and already known associations between vasohibin dysfunction and cancer [251,252].

A high ∆2 α-tubulin level in non-small-cell lung cancer (NSCLC) cells was associated with shorter overall patient survival and resistance to vinorelbine [211]. On the contrary, ∆2 α-tubulin was undetectable in prostate cancer cell lines (LNCaP and PC3), but was present in control cells [246].

Tubulin acetylation is associated with several types of cancer. An increased level of acetylation was reported in head and neck squamous cell carcinoma, for which it can be used as a prognostic marker [253]. An elevated level of tubulin acetylation in breast cancer cell line (MCF-7) is associated with the development of colchicine-resistance [254]. Additionally, a higher level of acethylated tubulin in primary breast tumors is linked to the basal-like subtype of breast cancer, in which it promotes adhesion and invasion of breast cancer cells, increasing the risk of disease progression and death [255]. Overexpression of ATAT1 in cultured nonmetastatic lines of breast cancer cells promoted the formation of microtubule-based membrane protrusions, structures characteristic of metastasis [255]. The level of microtubule acetylation was also shown to affect epithelial–mesenchymal transition and cell polarity [256].

Recent studies provide evidence that phosphorylation of serine 21 of HDAC6 by G protein-coupled receptor kinase 5 (GRK5) promotes deacetylase activity in ovarian (HeLa) and breast (MDA MB 231) cancer cell lines. An increased level of acetylated α-tubulin sensitizes these cells to the anti-apoptotic activity of paclitaxel [257]. The high expression of HDAC6 was also linked to poor prognosis of oral squamous cell carcinoma (OSCC) [258], oncogenic transformation [259], and EMT [260].

**Table 3:** Alterations in tubulin post-translational modifications (PTMs) in cancer.

PMT	Changes	Cancer	Outcome	Reference
α-, βIII-, βIV-tubulin tyrosination	elevated level	breast cancer cell lines	paclitaxel resistance	[261]
α-tubulin detyrosination	*TTL* down regulation	breast cancer lines,	increasing metastasis and tumor aggressiveness	[245]
		non-epithelial tumor of different origin	tumor growth correlates with loss of TTL activity	[262]
		primary neuroblastomas	impaired neuronal differentiation and poor prognosis	[248]
∆2 α-tubulin	elevated level	prostate cancer cell lines	n/a	[246]
		non-small-cell lung cancer	poor outcome, vinorelbine resistance	[211]
		breast cancers	high aggressiveness and poor prognosis	[247]
∆2 β IVb-tubulin		hepatic carcinoma (rat)	increased in cancer with respect to healthy liver	[263]
α-tubulin acetylation	HDAC6 knockdown	ovarian, breast epidermoid carcinoma cell lines	mitotic arrest, and cell death	[259]
	HDAC6 inhibition	nsclc cell lines	marker of better prognosis	[264]
	HDAC6 overexpression	breast cancer	good prognosis	[265]
		oral squamous cell carcinoma	correlates with tumor stage	[258]
	MEC-17 overexpression	lung cancer animal model	cancer cells migration and facilitated invasiveness	[256]
	MEC-17 downregulation		higher tumor grade	[256]
	elevated level of tubulin acetylation	head and neck cancer	correlates with tumor grade	[253]
		primary breast tumors	correlates with metastatic phenotype	[255]
		breast cancer cell lines	colchicine-resistance	[254]
		ovarian and breast cancer cell lines	paclitaxel sensitivity	[257]
Glutamylation	elevated levels	prostate cancer cells	n/a	[246]
		breast cancer cell lines	colchicine-resistance	[254]
Glycylation	TTLL3 downregulation	colorectal cancer	risk factor for carcinoma development	[266]
Phosphorylation of α-tubulin (Ser 165)	dephosphorylated (S165D) α-tubulin	breast cancer cell lines	hyperproliferation and increased metastatic potential	[267]

Phosphorylation of α-tubulin at Ser 165 residue by protein kinase C, in turn, stimulates microtubule dynamics in human breast cancer cells [78,79,267]. It seems that phosphorylation of α-tubulin at Ser 165 can act as a switch that controls the expression of EMT markers in nontransformed human breast cells and the rate of proliferation of breast tumors [79,267].

A few studies suggested that changes in the levels of tubulin glutamylation [246] and glycylation [266] are observed during tumorigenesis. Some unusual post-translational modifications have been detected in lung and hepatic cancers. The removal of the final two residues of the β IVb-tubulin C-terminal tail was identified in more advanced stages of liver cancer and metastasis to lung in a rat model of hepatic carcinoma [263].

### 5.3. Microtubule-Associated Proteins and Cancer

The data concerning MAPs in cancer are limited. Up to now, only a few MAPs, including Stathmin-1, EB1, CLIP-170/CLIP1, and some structural MAPs, have been associated with cancerogenesis, outcome prognosis, and chemotherapy sensitivity [268,269,270,271].

As already mentioned, Stathmin-1 was originally identified as an oncoprotein. Elevated levels of Stathmin-1 is a poor prognostic factor in many cancers, including leukemia, prostate, breast, lung, ovarian, cervical, endometrial, oral nasopharyngeal gastric, and colorectal cancers [272,273,274,275,276,277,278,279,280]. Some studies suggested that the silencing of the Stathmin-1-encoding gene can inhibit cancer cell migration and metastatic potential [281]. The data concerning correlation of the level of Stathmin-1 expression and resistance of cancer to chemotherapy are contradictory. Several studies show that Stathmin-1 overexpression increases the sensitivity of breast and lung cancer cells to taxanes and/or vinca alkaloids [282,283]. However, in epithelial carcinomas, nasopharyngeal carcinomas, breast cancer, and esophageal squamous cell cancer, the increased taxane sensitivity was correlated with Stathmin-1 silencing [284,285,286,287,288]. Interestingly, not only the protein level but also its phosphorylation state was correlated with cancerogenesis and drug resistance [289].

Increased levels of EB1 and CLIP-170/CLIP1, two +TIP proteins, enhances paclitaxel sensitivity in breast cancer cell lines and the response to taxane-containing therapy in patients [290,291]. On the other hand, a decrease of CLIP-170/CLIP1 expression correlates with patients survival in the case of glioma [292].

Structural MAPs have also been related with carcinogenesis. The expression of neuronal MAPs, Tau, MAP2, and MAP4 was detected in non-neuronal cancer tissues. For example, Tau overexpression observed in breast and ovarian cancer cells was correlated with a poor outcome [293,294,295], while downregulation of Tau in breast and ovarian cancer cell lines increased sensitivity to paclitaxel [293,295]. Because Tau and taxanes bind to the same tubulin surface, it was proposed that Tau may compete with paclitaxel for binding to β-tubulin, causing taxane ineffectiveness [293,295]. On the other hand, in mice, docetaxel-sensitive pancreatic neoplasms show a higher level of Tau and MAP2 with respect to those that are docetaxel-resistant [296,297].

MAP2 was proposed as a diagnostic marker in pulmonary neuroendocrine carcinomas, some non-small-cell lung carcinomas [298], Merkel cell carcinomas [299], and oral squamous cell carcinoma [300], but not in metastatic melanomas (while abundant in primary melanomas) [301]. Overexpression of MAP2 in melanoma cell lines leads to microtubule stabilization, associated with G2–M phase cell cycle arrest, growth inhibition, and cancer cell apoptosis, both in vitro and in a nude mouse model [301,302]. Decrease of MAP2c accompanied with a decrease in βIII-tubulin expression was also observed in vinca-resistant neuroblastoma cell lines [303].

Recently, also, MAP1B was shown to be expressed and a marker of a poor outcome in urothelial cancer [270]. Silencing of MAP1B in urothelial cancer cell lines decreased the cell migration and invasiveness [270].

An elevated level of MAP4 and resistance to vinca alkaloids have been observed in childhood acute lymphoblastic leukemia (ALL) cells [304], while leukemia cell lines resistant to the epothilone and hypersensitive to microtubule-destabilizing agents increased the levels of both MAP4 and βIII tubulin [305]. In esophageal squamous cell carcinoma, an increased level of MAP4 was shown to be a poor outcome marker, and its intratumor silencing inhibited cell growth in nude mice [306]. Very similar observations were also made in lung adenocarcinoma [307].

An increased level of MAP7 is a marker of poor prognosis in leukemia and cervical cancers [269,308,309]. Moreover, it was shown that MAP7 promotes migration and invasiveness of cervical cancer cell lines by inducing EMT transition [309].

## 6. Conclusions and Perspectives

Microtubule dynamics play a key role in the proper execution of cell division. Thus, it is not surprising that a large number of MTAs have found application as clinical drugs against different types of cancers. Unfortunately, MTAs are also toxic to healthy tissues. Therefore, reducing the toxicity of anticancer MTAs and understanding the causes of cancer cell resistance are extremely important. The main direction of research worldwide includes: (i) a comprehensive understanding of the tubulin code in cancer cells and the selective manipulation of tubulin isotype expression [121], (ii) an improvement of the potency of drugs and increased tumor specificity [18], (iii) combination therapy, with nanoparticles and anticancer drugs working synergistically to delay the onset of drug resistance [310], and (iv) the use of antibody–drug conjugates (ADCs) as a potent class of anticancer therapeutics that confer selective and sustained cytotoxic drug delivery to tumor cells.
